# Development of a Mouse Model of Abdominal Cutaneous Flaps for Breast Reconstruction

**DOI:** 10.1371/journal.pone.0052829

**Published:** 2013-01-07

**Authors:** Daniel John Womac, Arun Prathap Palanisamy, Rene Eslick, Dennis Kenneth Schimpf, Kenneth David Chavin

**Affiliations:** 1 Divisions of Transplant Surgery, Department Of Surgery, Medical University Of South Carolina, Charleston, South Carolina, United States of America; 2 Divisions of Plastic Surgery, Department Of Surgery, Medical University Of South Carolina, Charleston, South Carolina, United States of America; Harvard Medical School, United States of America

## Abstract

Autologous tissue transfer, in addition to replacing tissue that was lost during injury or surgery, offers women an excellent option to improve cosmetic appearance and self-confidence following mastectomy due to breast cancer. However, flap necrosis is a complication in obese patients undergoing this procedure. We created a mouse model to study the flap-related complications that leads to decreased flap survival in autologous breast reconstruction.

**Methods:**

Left superficial inferior epigastric (SIE) pedicle abdominal-cutaneous flaps were elevated in 8 week-old, obese ob/ob male mice and their lean littermates. Flaps were followed by serial photography. Area of flap necrosis was measured at 7 days. Statistical analysis was performed.

**Results:**

Necrosis was observed at the distal margin of the flaps, in both lean and obese groups. Lean left SIE flaps (n = 8) had a total area flap necrosis of 9.1% at 7 days whereas obese left SIE flaps (n = 8) had a total area flap necrosis of 45.5% at 7 days. Obese flaps had a statistically significant increase in necrosis compared to the lean flaps, p = 0.001.

**Conclusions:**

There was a significant difference between flap survival in lean and obese SIE pedicle flaps in our mouse model. We have developed the first flap model of obesity utilizing the superficial epigastric pedicle in the mouse. This model is optimal for future studies to dissect out mechanisms that lead to the complications related to flap survival for breast reconstruction, especially in obese subjects.

## Introduction

It is estimated that nearly half of the patients diagnosed with breast cancer in the United States will undergo mastectomy as their primary treatment [1]. Autologous tissue transfer offers women an excellent option to improve cosmetic appearance and self-confidence after mastectomy. Fasciocutaneous flaps as rotational or perforator flaps has been increasingly utilized. The muscle sparing Transverse Rectus Abdominus Muscle (TRAM) or Deep Interior Epigastric Perforator (DIEP) flaps are great options for patients seeking autologous breast reconstruction after mastectomy.

The incidence of obesity continues to increase in the United States and elsewhere reaching epidemic proportions. Obesity is a risk factor for more severe stages of cancer necessitating a larger tissue defect [2], [3], [4], [5]. This leads more women to seek autologous tissue transfer for breast reconstruction. However, flap necrosis in autologous breast reconstruction is a complication more often seen in obese patients [6], [7], . A better understanding of the injury process using lean and obese animal models would prove to be beneficial for developing future treatment options.

The laboratory rat is a well-documented experimental model for studying abdominal flaps used for breast reconstruction [10], [11], [12]. The rat is physically larger than the mouse, has thicker skin, and larger vessels. Our group has previously developed a obese rat model for studying abdominal flaps [13]. The laboratory mouse, however, has numerous advantages over rat as a model to study the molecular mechanisms involved during disease progress; i) mouse has a greater genetic diversification, ii) offer the ability to study the biochemical processes of disease by use of knockout technology, iii) better availability of specific tools to study the molecular mechanisms of disease (ex. Antibodies and other reagents tailored for mouse samples), and iv) relatively less expensive than other mammalian models. In addition, there are no studies describing abdominal fasciocutaneous flaps in mice utilizing the superficial inferior epigastric (SIE) vascular bundle. The superficial inferior epigastric artery supplies blood to the abdominal wall skin, subcutaneous tissue and fat and underlying fascia. The SIE vascular bundle can be compared to the superficial inferior epigastric pedicle (SIEP) flaps used in obese patients, as well as mimic TRAM or DIEP flaps.

Given these compelling advantages, we sought to develop a mouse model of abdominal-cutaneous flaps, to mimic autologous breast reconstruction. A second aim of this study was to assess the influence of obesity on flap survival. Understanding the mechanisms of flap necrosis in a mouse model can lead to the future development of therapeutic interventions in humans.

## Materials and Methods

### Ethics Statement

The use of animals is necessary in this study because of the nature of information sought. All rodents used for surgeries were initially anesthetized using isoflurane in desiccators then followed by isoflurane as needed. Animals were observed post-operatively for signs of distress as in respiratory distress, blood pressure, and discernable pain. Buprenorphine was given as an analgesic drug to reduce pain and discomfort. Animals are removed from the study and euthanized by by exsanguination (under anesthesia) or CO2 when clearly suffering negates the need to continue humanely in accordance with the Medical University of South Carolina's Institutional Animal Care and Use Committee (IACUC) policy. This study was reviewed and approved by the Medical University of South Carolina's IACUC (AR# 2175: Effects of Obesity on Musculocutaneous Flap Survival and Ischemia-Reperfusion Injury).

### Animals

Nine-week-old male ob/ob mice and their lean littermates (C57BL/6) (The Jackson Laboratory, Bar Harbor, Maine) were used. Animals were kept at a constant temperature of 37 degrees Celsius, during and after all surgical procedures by use of heating pads. All animals were housed individually in the animal facilities of the institution. Animals were fed ad libitum with standard pellet type chow. Animals were housed in conditional temperature, humidity, and photoperiods (12-hour light/12-hour dark cycles) with staffed watering and feeding in keeping with IACUC policy.

### Flap Design and Surgical Methods

**Figure 1:**
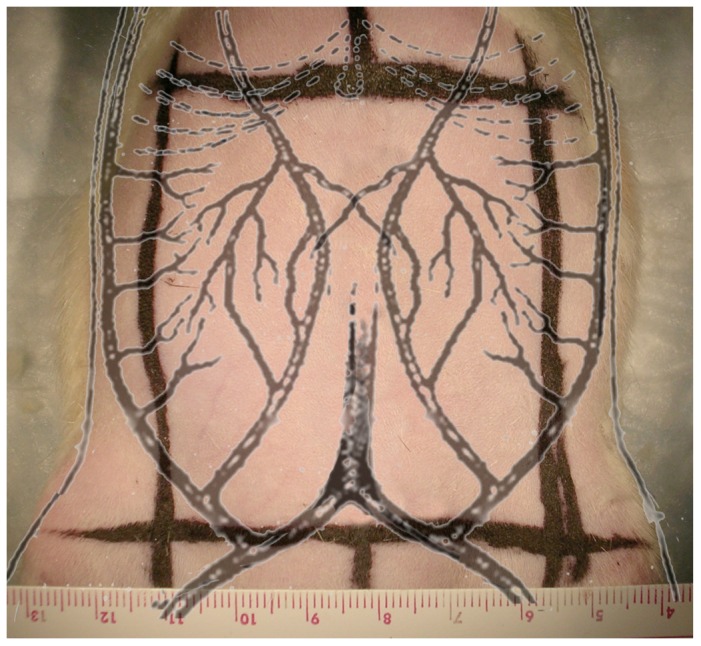
Outline of the surgical borders and blood supply of the abdominal cutaneous flap.

**Figure 2:**
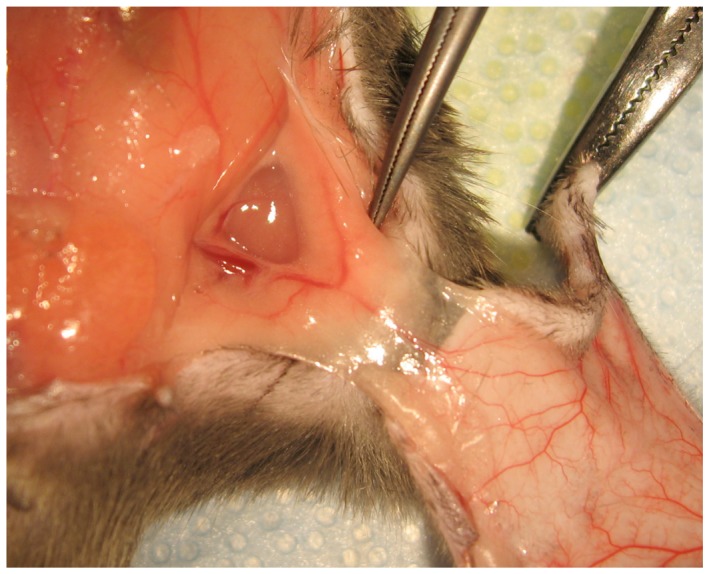
Operative image of the SIE pedicle and island skin flap.

The ob/ob mice were phenotypically obese, based on weight and visual appearance, compared to the lean mice. All mice were anesthetized with intraperitoneal injection of pentobarbital (concentration = 1∶10; dose = 0.01 mL/gm). The anterior abdominal wall was shaved with clippers, prepared with 70% isopropyl alcohol and betadine, and draped in a sterilely. An abdominocutaneous flap based off the left SIE artery was elevated using dissecting scissors. Anatomical landmarks were used to determine the flap design. The superior margin was a horizontal line crossing costal margins from left to right sides just caudal to the xiphoid. The inferior border was a horizontal line crossing the midline from left anterosuperior iliac spine to the right. The lateral borders were determined by vertical lines from the anterior axillary folds crossing the superior and inferior lines (Figure 1). Elevated flaps contained skin and subcutaneous fat and fascia. Abdominal wall musculature was left intact. The right SIE vessels and both superior epigastric bundles were identified and divided. Furthermore, all perforators and the lateral branch of the left SIE artery were divided, leaving the sole blood supply to the entire flap from the medial branch of the left SIE vessels (Figure 2). After elevation of the flap, the tissue was inserted using running 5-0 prolene suture. On post-operative day 7, all flaps were photographed, collected and stored in either 10% paraformaldehyde for histology or were flash frozen in liquid nitrogen and stored at −80 degrees Celsius for subsequent biochemical analysis. Following flap sample collection, all animals were euthanized by isoflurane overdose and cervical dislocation.

The operative time taken is approximately 30 minutes per mice in all groups. All of the surgeries were performed by a PGY3 surgery resident in the lab. We did not run into any difficulties during surgery between the two groups of mice and we believe that surgical time did not affect changes in flap necrosis between both groups.

### Estimation of Flap Viability

Obese mice and their lean littermates underwent flap elevation and closure as described above. Post-operatively, mice were photographed at day 0 and 7 using a Canon® SD700 IS digital camera. Using the images from post-operative day 7, percentage of flap necrosis and viability was determined by area measurements using NIH Image J software. Percentage of flap area necrosis was measured by dividing the necrotic area of the flap by the total area of the flap, then multiplying it times 100. (Percent Flap Necrosis = Necrotic Flap Area/Total Flap Area×100).

### Statistical Analysis

Because all statistical comparisons were made between two mice groups, lean and obese, a Student's two-tailed *t-*test was used to assess differences in flap viability. All results are presented as the mean ± standard deviation. *P* values less than 0.05 were considered statistically significant.

## Results

**Figure 3:**
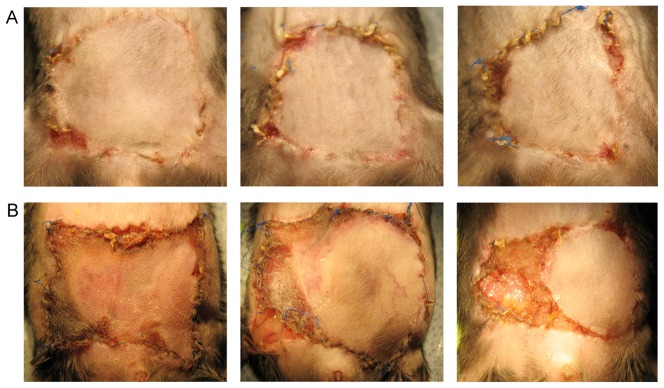
Post operative day 7 images of lean and obese SIEP flaps. (A) Lean SIEP flaps. (B) Obese SIEP flaps. Note that all necrosis seen is consistently contralateral and most distal to the blood supply.

A total of 18 animals, 8 lean (mean age 62 days; mean weight, 25.41 gm) and 10 obese (mean age, 64 days; mean weight; 38.72 gm), underwent flap elevation and closure (Table 1). Flap necrosis was visualized on day 3 and photographed on day 7. There was marked increase in necrosis in both groups between days 3 and 7. The majority of necrosis took place along the right and superior borders, contralateral and distal to the blood supply, which remained consistent throughout the experiments. Obese mice had more pronounced necrosis visually (Figure 3, bottom panel), as compared to the lean (Figure 3, top panel). Of the 18 animals, only 2 had complete flap loss (11.1%), a success rate of 88.9%. The two flaps lost were auto cannibalized by the obese mice.

**Table 1:** Age, weight and sample size of each phenotypical mouse used for flap analysis.

Phenotype:	Lean	Obese
Mean Age	62 days	64 days
Mean Weight	25 g	39 g
Sample Size	N = 8	N = 8

**Figure 4:**
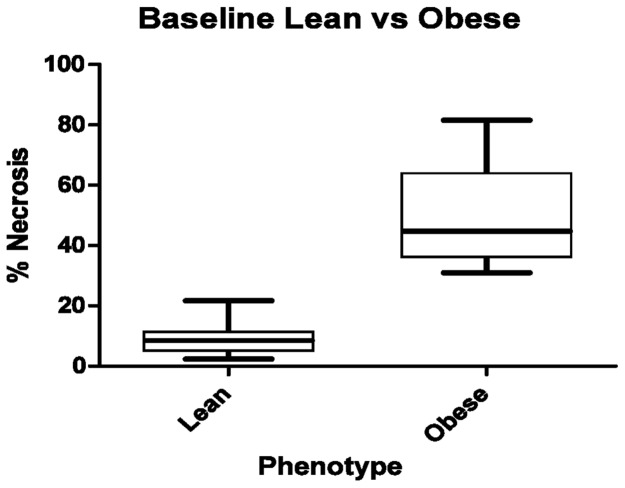
Mean percent area of flap necrosis at post operative day 7. Obese (n = 8), 45.51±17.03%, and lean (n = 8), 9.91±6.03. (P<0.001).

The two mice with complete flap loss were not included in measurements of lean versus obese flap necrosis, since the degree of necrosis cannot be quantified. Quantification of flap necrosis on day 7 showed a markedly increased amount of flap necrosis in the obese group (n = 8) compared to the lean (n = 8). The mean percent area of flap necrosis at 7 days was 9.91±6.03% in the lean group compared with 45.51±17.03% in the obese group (P<0.001), an absolute difference of 35.6% (Figure 4).

## Discussion

In this study, we sought to develop an abdominal fasciocutaneous flap in a mouse model to further investigate the effects of obesity as it pertains to flap survival in autologous tissue breast reconstruction. There are numerous studies on rats involving surgical abdominal flaps, however, very few in the mouse. The laboratory mouse is an excellent experimental animal. They have a relatively low cost, a diverse amount of genetic alterations, and the ability to perform specific gene knockouts to further explore the mechanisms behind pathologic events. We used B6.V-Lep ob mice, more commonly known as ob/ob mice, to study the effects of obesity on flap survival. The B6.V-Lep ob mouse has spontaneous genetic mutation which leads to a lack of functional leptin, the hormone that controls satiety. The mice suffer from hyperphagia, insulin resistance and clinical obesity [14], [15], [16]. We chose 9 week old animals that were phenotypically obese, however had not reached the age of developing diabetes and vasculopathy [16].

Although there are studies related to flaps in the laboratory mouse [17], [18], [19], there have been no studies related to abdominal fasciocutaneous flaps as it pertains to breast reconstruction and/or obesity. Talidede et al. concluded that the dorsal lateral thoracic artery pedicle island skin flap in the C57BL/6 mouse lead to reproducible results of flap necrosis, and further concluded that preconditioning the flap to ischemia reduced flap necrosis [17]. Losken et al. describes myocutaneous rectus abdominus flaps which had 93% complete flap survival [18]. We found similar results in our experiments, with 89% of the flaps surviving to day 7. These studies however did not develop a model that mimics the obese patient.

The use of pedicled flaps in a rat model dates back to 1965 when McFarlane *et al.* designed a pedicle flap to study necrosis [20]. Later, Finseth and Cutting studied pedicled flaps and demonstrated that consistent flap necrosis occurred contralateral to the neurovascular pedicle [21]. Padubidri and Browne performed SIE flaps in the rat, occluding the lateral branch of the SIE artery. Their results were more consistent than previous results [11]. A previous study conducted at our laboratory used the same flap design as Padubidri and Browne, to compare flap viability in fatty and lean Zucker rats. We have previously demonstrated that obesity lead to decreased flap survival using an abdominal fasciocutaneous SIE pedicle flap in a rat model, and that obese rat tissue had decreased ATP content compared to the lean rats [13]. Consensus among investigators is that obesity has a negative effect on flap survival in human subjects. Retrospective reviews analyzing outcomes in patients who underwent free or pedicled TRAM flap breast reconstruction have supported this by demonstrating that obese patients were likely to develop post operative complications more so than non-obese patients [6], [7], [8], [9], [22], [23].

A concern with this model is that the SIEP flap is known to be a less reliable pedicle for abdominal tissue in humans when compared to the TRAM and DIEP flaps, which are generally based on the deep systems. For this reason most reconstructive surgeons choose the deep systems for flap harvest to decrease chances of complications including fat and skin necrosis especially in the obese patient. At our institution we do approximately 120 perforator flaps a year and less than 5 of those flaps per year are based on the superficial epigastric system. The epigastric system is unreliable at times or non-existent in some human patients. We have found in the mice model that this system appears to be the dominant system. There are little to no significant deep perforating branches present on the mouse model when raising the flaps. However, we feel that the more important issue is that the anatomical structure, as well as the physiological basis of the superficial epigastric based flap in mice is highly similar to the DIEP flaps in humans. The fact that these flaps are both composed of fat and skin only, and based on an arterial and venous pedicle, it is reasonable to assume these flaps are very similar in there characteristics and behavior. Therefore this mouse model would seem reasonable to use as a model of the DIEP flap for breast reconstruction.

Our results demonstrate a reliable and reproducible mouse model of abdominal flaps used for breast reconstruction. Flap necrosis occurred furthest away from the blood supply and was consistent from mouse to mouse. The ease of the procedure and animal up keep is relatively simple. Furthermore, our results demonstrate that obesity leads to increased flap necrosis when compared to lean. There was a statistically significant difference in flap necrosis between the obese and lean groups at 7 days. These results are similar to our previously published work on rats, where we further concluded that obese rat tissue had less ATP content than the lean^21^. Future studies will entail histological and biochemical analyses of the mouse flap tissue and to initiate therapeutic interventions as well as use knockout mouse to further determine the mechanisms behind flap necrosis in obese animals.
